# Knockout, Knockdown, and the Schrödinger Paradox: Genetic Immunity to Phenotypic Recapitulation in Zebrafish

**DOI:** 10.3390/genes15091164

**Published:** 2024-09-03

**Authors:** Álvaro J. Arana, Laura Sánchez

**Affiliations:** Departamento de Zoología Genética y Antropología Física, Facultad de Veterinaria, Universidade de Santiago de Compostela, Campus de Lugo, 27002 Lugo, Spain; alvaro.arana@usc.es

**Keywords:** maternal contribution, phenotypic variability, zebrafish development, knockout, knockdown, genetic compensation response, nonsense-mediated decay, morpholino oligos, CRISPR/Cas9, gene function

## Abstract

Previous research has highlighted significant phenotypic discrepancies between knockout and knockdown approaches in zebrafish, raising concerns about the reliability of these methods. However, our study suggests that these differences are not as pronounced as was once believed. By carefully examining the roles of maternal and zygotic gene contributions, we demonstrate that these factors significantly influence phenotypic outcomes, often accounting for the observed discrepancies. Our findings emphasize that morpholinos, despite their potential off-target effects, can be effective tools when used with rigorous controls. We introduce the concept of graded maternal contribution, which explains how the uneven distribution of maternal mRNA and proteins during gametogenesis impacts phenotypic variability. Our research categorizes genes into three types—susceptible, immune, and “Schrödinger” (conditional)—based on their phenotypic expression and interaction with genetic compensation mechanisms. This distinction provides new insights into the paradoxical outcomes observed in genetic studies. Ultimately, our work underscores the importance of considering both maternal and zygotic contributions, alongside rigorous experimental controls, to accurately interpret gene function and the mechanisms underlying disease. This study advocates for the continued use of morpholinos in conjunction with advanced genetic tools like CRISPR/Cas9, stressing the need for a meticulous experimental design to optimize the utility of zebrafish in genetic research and therapeutic development.

## 1. Introduction

The zebrafish (*Danio rerio*) has established itself as a fundamental model for studying the genetic control of vertebrate development due to its favorable biological characteristics, including high fecundity, transparency, and rapid external embryo development [1]. Zebrafish reach sexual maturity at three months, similar to mice, but their embryonic development is much faster, with most organs and glands formed within five days post-fertilization (dpf) [2]. Zebrafish share about 70% of their genes with humans, including 84% of known human disease genes, with well-conserved structures like the neuroendocrine system [3,4]. The availability of extensive public databases like NCBI, Ensembl, and ZFIN further enhances their utility in genetic research and the study of human diseases.

Genetic approaches in zebrafish, such as classical and reverse genetics, have greatly advanced our understanding of gene function. Classical genetics initially relied on mutagenesis with agents like N-ethyl-N-nitrosourea (ENU) to induce random mutations and identify genes responsible for specific phenotypes. [5]. Techniques like morpholinos (MOs) [6,7], and more recently, targeted gene editing tools such as CRISPR/Cas9, ZFN, and TALEN, have become essential in this research [8]. However, these methods have also revealed significant phenotypic discrepancies between knockout mutants and morphants, leading to debates regarding the validity of the results produced by different techniques. Two key factors that contribute to these discrepancies are genetic compensation and maternal contributions. Genetic compensation, through mechanisms like the genetic compensation response (GCR), allows mutants to upregulate compensatory genes, potentially masking expected phenotypes [9]. Additionally, the maternal contribution of RNA and proteins during early development can maintain normal gene function despite the absence of zygotic mRNA, further complicating phenotype manifestation [10].

These complexities are especially evident in CHARGE syndrome (MIM# 214800), a genetic disorder mainly linked to mutations in the *CHD7* gene [11]. *CHD7* encodes a protein crucial for chromatin remodeling, which governs DNA accessibility and the regulation of key developmental genes. The significant phenotypic and genetic variability seen in this syndrome highlights the unpredictable nature of genetic outcomes, making it essential to consider factors like maternal contributions, genetic compensation, and epigenetic modifications that influence gene expression [12,13,14,15,16,17,18,19,20]. This unpredictability, reminiscent of Schrödinger’s paradox, emphasizes the need for careful observation, as the genetic system may remain in a “superposition state” until directly observed, complicating our understanding of gene function and expression.

## 2. Techniques for Studying Human Genetic Diseases in Zebrafish

The study of human genetic diseases has advanced significantly due to the evolution of various genetic techniques. These tools not only aid in identifying the genes involved in various pathologies but also provide deeper insights into how these genes function and influence human biology. Among the most impactful methodologies are forward genetics, reverse genetics, and modern gene editing techniques. However, the application of these techniques has also sparked debates, particularly concerning phenotypic discrepancies observed between different approaches.

### 2.1. Forward Genetics

Forward genetics has been crucial in the early stages of genetic research, particularly in model organisms like zebrafish. This approach involves inducing random mutations and then screening for phenotypic changes to identify genes linked to specific traits or diseases. Early studies used mutagens such as γ rays [21,22] and ENU [23,24], with ENU being especially effective due to its high mutation rate in both post-meiotic and pre-meiotic germ cells [1,5]. Zebrafish are ideal for these studies, thanks to their key characteristics that support complex and extensive phenotype screening and genetic mapping. With over 40,000 mutant alleles generated, this approach has been fundamental in understanding approximately 60% of protein-coding genes in this species.

Besides chemical mutagens like ENU and radiation, random insertional mutagenesis using viral vectors such as retroviruses and transposons has also been used. These methods randomly insert DNA into the genome, disrupting genes and creating mutations that are essential for identifying genes critical to vertebrate development and disease models [25,26,27,28,29]. However, these techniques come with challenges. Positional cloning to identify mutations is costly and labor-intensive [30,31]. Random mutagenesis often produces heterozygous mutants, complicating the identification of recessive phenotypes, and methods like ENU do not cover the entire genome, limiting the scope of genetic screens [32]. Furthermore, ENU mutagenesis introduces multiple genetic variants within the genome, potentially creating a “genetic burden” where the background is polluted with additional mutations. The consequences of this genetic load are not well understood and could influence the phenotypic outcomes or complicate the interpretation of results [1,5,21,22,23,24,33,34]. Given these limitations, the emergence of precise genome editing tools such as TALEN, ZFN, and CRISPR/Cas9 has revolutionized the field, enabling more accurate and targeted genetic modifications [35,36,37].

### 2.2. Reverse Genetics

Reverse genetics is a foundational tool in functional genomics, enabling researchers to investigate gene functions by either knocking down or knocking out specific genes. Gene knockdown involves reducing gene expression to study the resulting phenotypic changes, while gene knockout entails completely disabling a gene to observe its effects on the organism. These strategies are pivotal in unraveling the roles of individual genes, particularly within complex developmental processes. This approach gained significant momentum in the early 21st century, driven by the rapid expansion of genomic data and the need for targeted gene studies [3,38,39].

#### 2.2.1. Knockdown and MOs

In zebrafish research, morpholinos (MOs) are widely used for gene knockdown, allowing the study of specific gene functions during critical developmental stages. MOs are synthetic oligonucleotides that inhibit gene expression by either disrupting mRNA splicing or blocking translation. The latter is particularly important when targeting maternal mRNA, which plays a crucial role in early development. MOs have been instrumental in exploring gene functions in zebrafish, such as the role of *chd7* in CHARGE syndrome [40,41,42]. However, MOs are temporary and prone to off-target effects, making strict adherence to experimental protocols essential. These off-target effects often manifest as reduced head and eye size, notochord malformations, and craniofacial defects, primarily due to *p53*-mediated apoptosis, which can be partially mitigated by co-injecting a *p53*-targeting morpholino [43,44,45]. To minimize these effects, important guidelines include dose-dependent administration, the use of *p53*-targeting MOs, and the co-injection of wild-type (WT) mRNA for phenotype rescue [46,47,48,49,50].

Although MOs have been used for gene knockdown in zebrafish, their use has generated controversy due to off-target effects and often inconsistent phenotypic correlation with mutants [1,5,8,25,43,44,45,51,52,53]. Multiple studies, including Kok et al. (2015), have shown that MOs often produce more severe defects than knockouts, with 80% of morphants displaying phenotypes not observed in mutants [9,10,27,54,55]. These discrepancies are frequently attributed to improper MO dosing, transcriptional adaptation, and maternal RNA contributions, leading to a high rate of false positives. Validating these knockdowns is further complicated by challenges in assessing protein loss, especially in zebrafish, where the limited availability of species-specific antibodies makes it difficult to confirm complete protein knockdown. Even minimal residual protein can obscure phenotypic effects, complicating dose adjustments and result interpretation. Lawson (2016) highlighted specific genes such as *tcf7***,** *ift88*, and *wnt1*, where morphants exhibited anomalies due to off-target effects or p53-mediated apoptosis. The discrepancies between morphants and mutants are primarily due to genetic compensation and transcriptional adaptation, which allow organisms to mitigate gene loss [55]. 

#### 2.2.2. Knockout and CRISPR/Cas9

The advent of CRISPR/Cas9 technology has revolutionized reverse genetics by providing a more precise and reliable method for targeted DNA editing, making it the preferred approach for generating mutant zebrafish lines and other model organisms [35,56,57,58]. This technology is used in two main ways: stable knockouts and transient knockouts (CRISPants). Stable knockouts involve injecting sgRNAs and Cas9 into one-cell embryos, producing mosaic F0 founders. These F0s are bred to generate heterozygous F1 progeny, which are then crossed to obtain stable homozygous F2 mutants. This process typically takes over 4–6 months, enabling the study of gene function across generations and the long-term effects of gene disruption [35]. On the other hand, transient knockouts, or CRISPants, allow for rapid phenotype observation in injected F0 mosaic embryos, generating biallelic mutations in a high proportion of cells, enabling researchers to detect gene effects within hours or days [59,60,61,62,63,64,65].

Despite its advantages, CRISPR/Cas9 is not without limitations. While it significantly reduces off-target effects compared to MOs and offers greater versatility, the consistency of phenotypes still depends on rigorous experimental design and strict adherence to guidelines. Additionally, significant phenotypic differences have been observed between CRISPants and stable mutants, likely due to the presence of genetic compensation in stable mutants that is absent in CRISPants [66,67,68,69]. Although CRISPR/Cas9 has demonstrated improved phenotypic consistency compared to older methods like ENU mutagenesis or TALENs [32,63,70], studies like those by Kok and Lawson included relatively few CRISPR mutants, suggesting the need for a broader application of this technology. This includes the further exploration of CRISPants to fully realize the potential of CRISPR/Cas9 in studying gene functions across different developmental stages.

Additionally, exon selection is critical in both knockdown and knockout studies, as not all exons are included in every transcript isoform, leading to phenotypic variability. In knockout studies, the creation of indels can affect pre-mRNA splicing, sometimes enabling partial protein function rescue, as seen in conditions like Duchenne muscular dystrophy and certain retinal disorders. In knockdown experiments, exons are typically chosen based on canonical isoforms, which may not account for all protein variants, further contributing to inconsistencies [29,71,72,73].

## 3. The Schrödinger Paradox in Zebrafish Models

Understanding the role of maternal contribution and genetic compensation is crucial in zebrafish genetic research, as these factors can significantly alter phenotypic outcomes and complicate the interpretation of results from various genetic techniques. The complex interplay between maternal and zygotic gene products, along with compensatory mechanisms, highlights the importance of careful experimental design to accurately assess gene function and its relevance to human disease. The Schrödinger Paradox, a concept from quantum mechanics, underscores that gene expression may yield different phenotypes depending on experimental conditions, such as knockout versus knockdown techniques, challenging the traditional view of predictable, consistent outcomes.

### 3.1. Maternal Contribution

At the start of embryogenesis, the embryo relies entirely on maternal RNA and proteins provided during oogenesis for development until zygotic genome activation (ZGA). During the maternal–zygotic transition (MZT), these maternal products degrade, and zygotic instructions begin to take over, marking the shift to the zygotic phase [74,75,76]. Genetic and sequencing studies have indicated that the clearance of maternal transcripts is achieved through two sequential pathways [77,78,79], known as

1. Maternal Decay: Entirely mediated by maternal factors accumulated in mature oocytes.
2. Zygotic Decay: Dependent on de novo zygotic transcription products after fertilization.

**Figure 1:**
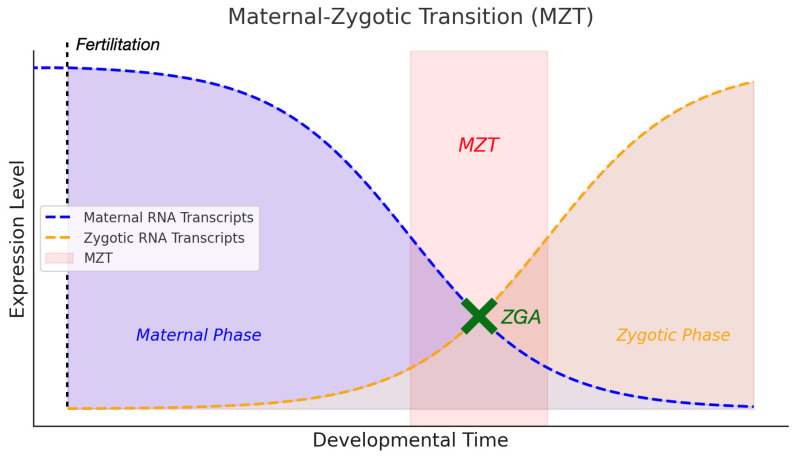
Changes in the expression of maternal and zygotic RNA transcripts during the MZT in embryonic development. This diagram illustrates the transition from maternal to zygotic control during embryogenesis in zebrafish. Before ZGA (green X), maternal RNA transcripts dominate, but their levels decrease as zygotic RNA transcripts increase post-ZGA, marking the shift in developmental control as the embryonic genome begins to take over from maternal factors. This figure represents the concept qualitatively and does not provide exact quantitative data. The figure helps illustrate how maternal mutations can obscure or alter phenotypic outcomes depending on their timing and persistence relative to ZGA.

In some maternal genes, an anticorrelation is observed where mRNA is rapidly degraded post-fertilization, while the proteins persist throughout the blastocyst stage [80]. The phenotypic outcomes in embryos can be influenced by mutations in the maternal genome rather than the embryo’s genome. For instance, maternal products can mask zygotic mutations, leading to variable phenotypes depending on the interplay between maternal and zygotic contributions (Figure 1). Figure 1 illustrates this transition, showing how maternal RNA transcripts dominate early development and are gradually replaced by zygotic transcripts post-ZGA, which is crucial for understanding the dynamics of maternal and zygotic mutants.

**Figure 2:**
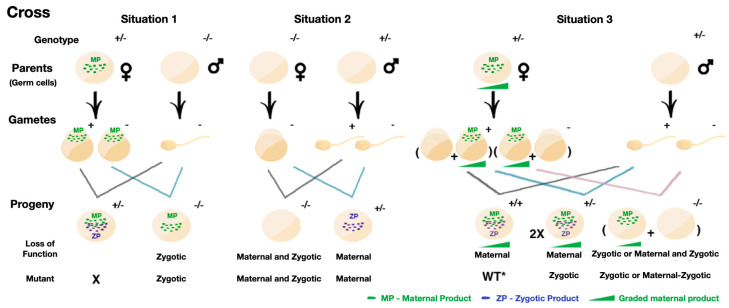
Maternal and zygotic contributions in early embryogenesis and the graded maternal contribution model. This figure illustrates three genetic scenarios that highlight the interplay between maternal and zygotic gene products during embryogenesis: **Situation 1:** A heterozygous (+/−) mother crossed with a homozygous recessive (−/−) father produces zygotic mutants. Here, MP can initially mask the zygotic mutation, but phenotypic defects emerge after ZGA. **Situation 2:** A homozygous recessive (−/−) mother crossed with a heterozygous (+/−) father leads to maternal or maternal–zygotic mutants. The absence of functional maternal products causes severe developmental defects from early stages. **Situation 3:** Involving two heterozygous (+/−) parents, this scenario introduces the concept of graded maternal contribution. The unequal distribution of maternal products among offspring results in a spectrum of phenotypes, from WT to zygotic mutants, and even mosaic phenotypes within the same organism. This concept is crucial in understanding genetic diseases and developmental processes, where the balance between maternal and zygotic inputs can either mask or amplify the effects of mutations. This figure underscores the importance of considering both maternal and zygotic contributions to fully understand gene function and its implications for development and disease. While graded maternal contribution could theoretically influence Situations 1 and 2, these scenarios are typically regarded as classical examples and are not expanded upon here. Abbreviations: MP, maternal product; ZP, zygotic product; WT, wild type.

Maternal effects generally emerge before zygotic transcription begins, particularly in species like zebrafish, where maternal products are crucial until zygotic genome activation (ZGA) [81,82]. While robust zygotic transcription typically starts after the mid-blastula transition (MBT), when cell cycles slow down [66], some genes begin transcription much earlier. Although the zebrafish MBT occurs at the 1000-cell stage, the primary transcription of certain genes has been observed as early as the 64-cell stage [83,84,85,86,87]. Following ZGA, which coincides with the 1000-cell stage, zygotic genes increasingly direct development [74,75,76,88]. The depletion of maternal products can lead to persistent phenotypes that last beyond the MZT and into adulthood, underscoring the enduring influence of maternal contributions [89,90,91]. Maternal–zygotic genes further complicate this dynamic, as mutations in these genes can affect both pre- and post-ZGA stages, with many mutants arresting development post-MZT due to inadequate maternal function [91]. Figure 2 illustrates the distinction between zygotic mutants, influenced by zygotic gene products (Figure 2, Situation 1) and maternal mutants, driven primarily by maternal gene products (Figure 2, Situation 2).

The Schrödinger Paradox illustrates how maternal contributions during the maternal phase can obscure the effects of zygotic mutations during the zygotic phase, leading to milder or unexpected phenotypes in mutants created by techniques like CRISPR/Cas9 [75,88]. For example, maternal cyclin-dependent kinase binding proteins in oocytes can mask phenotypes that would otherwise appear due to zygotic mutations [90]. This masking effect is central to the Schrödinger Paradox, where the presence or absence of gene products at critical stages can alter phenotypic outcomes. The degradation of maternal RNAs, regulated by microRNAs like *miR-430* in zebrafish, ensures the transition to zygotic control, but the persistence of maternal products can prevent expression of phenotypes linked to zygotic mutations [75]. After ZGA, during the zygotic phase, zygotic gene expression may compensate for deficiencies caused by maternal mutations. For example, in the zebrafish *pwg* mutant, severe defects in prechordal plate cell migration and anteroposterior axis extension are observed, but zygotic expression can partially compensate, allowing some development to continue. Similarly, in the *bsd* mutant, ventral caudal vein formation defects are mitigated by zygotic gene activation, aiding tissue repair and survival into adulthood [86].

Maternal CRISPants extend the utility of CRISPR/Cas9 by targeting genes with significant maternal contributions through various approaches. One effective method involves injecting CRISPR/Cas9 components into one-cell stage embryos to produce F0 individuals that are mosaics for the targeted gene. These F0 mosaics can transmit both the mutated gene and associated maternal products (such as RNA or proteins) to their offspring, enabling the F1 generation to inherit homozygous mutations and facilitating the identification of maternal-effect genes within a single generation [59,60,61,62,63,64,65]. This strategy not only accelerates research but also allows for the investigation of genes with critical maternal contributions that might be lethal in homozygous form, without the need for generating homozygous mutants across multiple generations [65].

### 3.2. Genetic Compensation

**Figure 3:**
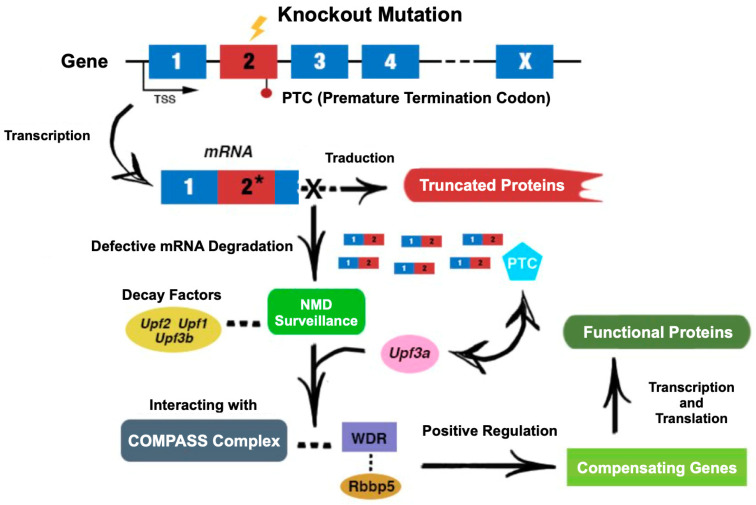
Schematic representation of the mechanisms underlying genetic compensation following a knockout mutation. The figure illustrates how a knockout mutation can lead to the formation of a PTC, which activates the NMD pathway. This pathway is crucial for degrading defective mRNA, preventing the production of truncated, non-functional proteins. The "X" in the figure indicates that these defective mRNAs are targeted for degradation via the NMD pathway rather than being translated. The NMD pathway not only eliminates these defective transcripts but also triggers the upregulation of compensatory genes that help restore functional protein levels. In zebrafish, mutations in specific genes can lead to the increased expression of related family members—a process dependent on the NMD pathway. Key components, such as UPF1, play a vital role in this mechanism by facilitating the degradation of mutant transcripts and enabling the activation of compensatory genes. Additionally, mRNA with PTCs can initiate genetic compensation through interactions between NMD factors, histone modifiers, chromatin remodelers, and components of the COMPASS complex, such as WDR5. These interactions enhance histone modifications, specifically H3K4me3, at the transcription start regions of compensatory genes, thus upregulating their expression. The transcription of mRNA with PTCs and sequence homology are essential for initiating this compensatory response. This figure is based on an original image from Rouf et al., 2023, but has been redrawn by the author [101].

Research in zebrafish has shown that phenotypic discrepancies between stable mutants and morphants often arise from off-target or toxic effects, particularly when using morpholinos (MOs). However, some stable mutants exhibit a GCR that is absent in morphants, which may explain why mutants often do not display severe phenotypic defects [54,69,92,93]. The GCR is a mechanism triggered by mutations that cause mRNA degradation through premature termination codons (PTCs). The Nonsense-Mediated Decay (NMD) pathway plays a crucial role in this process by eliminating defective transcripts and activating compensatory genes [94,95]. In zebrafish, mutations in genes such as *hbegfa*, *hif1ab*, and *vegfaa* lead to the upregulation of related genes in homozygous mutants—a response that heavily relies on the NMD (Figure 3). Additionally, the interaction between the NMD factor Upf3b and components of the COMPASS complex, such as WDR5, enhances histone modifications that activate these compensatory genes [95,96].

However, this compensatory mechanism is not always consistent; recent research indicates that GCR activation can vary with age, being present in older fish but absent in younger mutants [97]. This variability relates to the Schrödinger Paradox in genetic research, where phenotypic outcomes depend on experimental conditions, such as age or genetic background. For instance, CRISPR-generated embryos might initially exhibit defects that later normalize due to gene compensation, but some mutants that do not show early phenotypes might develop mild defects in adulthood [98,99]. This phenomenon helps explain why the *egfl7* gene causes vascular defects in morphants but not in mutants, as compensatory mechanisms like the upregulation of *emilin2* and *emilin3* can mitigate the loss of *egfl7* function [92].

The phenotypic differences observed between CRISPants and stable knockout mutants often result from the differential activation of GCR, which is not typically triggered in transient knockouts. While some stable knockout mutants fail to replicate the morphant phenotype, CRISPants generally show similar phenotypes, making them an efficient tool for in vivo gene function studies without needing stable mutant lines [65,66,67,68,69]. CRISPants, being mosaic mutants generated by CRISPR/Cas9 in the first generation, usually do not activate GCR because their mutations are temporary and partial, unlike the stable, homogeneous mutations seen in permanent mutants. For example, stable mutants of the *slc25a46* gene exhibit significant genetic compensation that alters gene expression—a response not observed in CRISPants. Additionally, the mosaic nature of CRISPants allows them to bypass early developmental lethality, enabling the study of gene function during later developmental stages [68,69,100].

These findings underscore the importance of gene families and transcriptional redundancy in early zebrafish development, which is closely linked to genetic compensation. Zebrafish, part of the diverse group of ray-finned fish, have retained about 20% of duplicated genes from a whole-genome duplication event around 350 million years ago [4,102,103,104]. This duplication, along with GCR, provides essential genetic robustness, crucial for survival and development when mutations occur in vital genes. However, this genetic complexity can lead to differential gene expression in mutants, significantly influenced by allele-specific expression. Such differences can result in misinterpretations of a mutation’s true impact, producing “red herrings” where genes near the mutation site appear differentially expressed due to genetic linkage rather than the mutation itself [105]. This issue, highlighted by the Schrödinger Paradox, challenges researchers to carefully discern which genes and pathways are genuinely affected by the mutation.

### 3.3. Quantum Phenotypes: Unraveling Maternal–Zygotic Interactions in Zebrafish

Genetic functions can be classified as strictly maternal or zygotic, depending on whether the mutant phenotype is determined solely by the genotype of the germline or the embryo, respectively. Maternal–zygotic interactions often reveal functional redundancy between maternal and zygotic gene products, emphasizing the gene’s sensitivity to dosage from both sources [106,107,108,109,110]. This is particularly evident in dominant maternal effects, where a phenotype manifests due to heterozygosity in a recessive gene, incorporating contributions from both maternal and zygotic genetics [110]. Such effects highlight situations where a WT offspring might exhibit a mutant phenotype because of maternal inheritance of certain genetic components, even if the offspring itself does not carry the mutation. Conversely, a mutant offspring might not show a phenotype if the maternal contribution compensates for the mutation. Experiments with CRISPR/Cas9 in *Drosophila melanogaster* further demonstrate this phenomenon, where maternal CRISPR components introduced into eggs led to mutations in offspring that did not possess the CRISPR genes, showcasing a non-Mendelian inheritance pattern driven by maternal contributions [110].

The concept of “quantum appearances” of phenotypes becomes particularly relevant in this context, as mutants with a specific genotype may sometimes exhibit a phenotype, while at other times, they do not. This variability can also be observed in WT organisms, complicating the interpretation of genetic studies. Figure 2, Situation 3, illustrates how the unequal distribution of maternal products and zygotic contributions leads to diverse phenotypic outcomes, underscoring the intricate interplay between maternal and zygotic effects in genetic research.

In mammals, maternal signals are crucial during early embryonic development, particularly at the two-cell stage, which coincides with ZGA and important developmental transitions, such as the mid-blastula stage in zebrafish. These stages involve significant changes in cell division and synchronization, triggered by reaching a critical nucleocytoplasmic ratio, at which point a proposed maternal cytoplasmic factor initiates transcription [87,105,111,112,113]. Although zygotic genes are often assumed to function independently, research in zebrafish and Xenopus has demonstrated that maternal gene products, including RNAs and proteins, are essential for early development and can mask or modify the effects of zygotic mutations. For instance, maternal–zygotic mutants for genes like *tcf7l1a*, *pou5f3*, and *chd* exhibit severe phenotypes, such as headlessness or extreme ventralization, that are not seen in purely zygotic mutants, highlighting the significant influence of maternal contributions [107,114]. Moreover, while zygotic genes like pdgfrb are thought to activate genetic compensation mechanisms in response to PTCs, these compensatory responses may often fail when maternal influences are present. This possibility is particularly suggested in maternal–zygotic genes like *chd7*, where most heterozygotes appear not to exhibit a phenotype, but genetic compensation in homozygous mutants may fail to rescue the phenotype and could affect different mutations unequally [115,116]. Mutations with PTCs might contribute to phenotypic variability, potentially impacting not only the mutants but also the WT siblings. The persistence of maternal gene products beyond the MZT could hypothetically lead to developmental anomalies and embryonic lethality in these maternal–zygotic genes.

The analysis of *nrp1a* and *pdgfrb* genes illustrates how genetic compensation in zygotic genes, which lack maternal expression, can obscure phenotypic outcomes—a phenomenon that can be considered another form of “quantum appearance”. For example, targeting *nrp1a* and *pdgfrb* through gene editing produced mutants that lacked the defects seen in morphants, suggesting that the phenotypes observed in morphants may result from off-target effects rather than genuine gene loss. However, recent findings indicated that *nrp1a* mutants exhibited significant phenotypes related to cardiac regeneration, including reduced angiogenic response and impaired epicardial regeneration, contrasting with previous observations showing no phenotype. This variability in phenotypic expression, akin to “quantum appearances”, highlights the necessity of using genetic mutants for more accurate and reliable results [8,117]. 

Gene editing techniques such as CRISPR/Cas9 and ENU-induced mutagenesis are crucial for generating mutations that impact gene function in different ways. CRISPR/Cas9 typically produces indels, resulting in frameshift mutations and PTCs that activate compensatory mechanisms like NMD and GCR, potentially mitigating harmful effects by upregulating homologous genes. Conversely, ENU primarily induces missense mutations that do not trigger NMD or GCR, which could lead to more severe phenotypes unless maternal contributions buffer these effects [93,94]. The *pdgfrb* gene also demonstrates significant phenotypic discrepancies between mutants generated through different techniques. For instance, an ENU-induced mutant with a point mutation exhibited distinct phenotypes compared to a TALEN mutant with a four-base deletion, likely due to genetic compensation by *pdgfra*, the paralog of *pdgfrb* [8,112]. These differences emphasize the need to consider the type of mutation and the potential for genetic compensation, as not all mutations produce observable phenotypes; some may remain silent or exhibit minimal effects. Additionally, the concept of “quantum appearances” of phenotypes complicates genetic interpretations, as conditionally exhibiting phenotypes can lead to variability in both mutants and wild types under different conditions.

## 4. The “Autoimmune” Genetic System of the Zebrafish

The newly proposed concept of the “autoimmune” genetic system in zebrafish emphasizes the heightened reactivity of the GCR mechanism and the graded contribution of maternal genetic material. The GCR mechanism relies on sequence homology, paralogy, and functional redundancy, along with the presence of mRNAs with PTCs, to target defective mRNAs for degradation [95,96,101], ensuring that mutations in mRNAs are quickly identified and degraded, thereby protecting the organism from potential harm and promoting healthy development. However, despite these surveillance systems in the nucleus and NMD, some PTC-bearing mRNAs can escape degradation and remain in the cytoplasm, where they may continue to participate in translation [118,119,120]. Because PTC-bearing mRNAs can escape the NMD pathway, we suggest that they might also evade clearance and exert effects during the zygotic stages. These mRNAs could continue to be translated, potentially leading to their degradation, and in genetically healthy organisms, this could result in toxic effects that disrupt normal development.

In fact, some mRNAs can escape maternal clearance through various strategies and have been observed to accumulate in later stages. Some RNAs have alternative processing pathways that do not depend on canonical miRNA mechanisms, as in the case of miR-21, which can be processed independently of the canonical Dgcr8 pathway [121]. Additionally, certain RNAs show elevated residual expression levels even when typical processing routes are disrupted, indicating the existence of less efficient backup mechanisms. Furthermore, RNAs can evade miRNA-mediated degradation through secondary structures that prevent miRNA binding or through interactions with proteins that protect them from degradation. The timing of RNA expression and its cellular localization can also influence its susceptibility to clearance, allowing RNAs expressed later or in cellular regions with lower concentrations of degrading enzymes to persist longer [122,123]. 

### 4.1. Concept of Graded Maternal Contribution

The concept of graded maternal contribution, introduced in this article, suggests that variability in the distribution of maternal gene products during gametogenesis and early embryonic development plays a crucial role in shaping the offspring’s phenotype, particularly in heterozygous organisms where homozygosity for a specific gene is lethal (Figure 2, Situation 3). These organisms possess one healthy and one mutated gene copy, with maternal contributions and zygotic function both playing vital roles. Contrary to the traditional view of a uniform distribution of maternal mRNA and proteins during meiosis, this concept proposes an uneven distribution of healthy and mutated mRNA among gametes and unequal partitioning during early cell divisions post-fertilization. This can result in phenotypic mosaicism, driven by the mosaicism of the maternal product among the blastomeres. In this scenario, different embryos may also exhibit varying phenotypes due to differing amounts of maternal genetic input. This variability, particularly significant before the MBT, can influence the development of complex traits and the expression of genetic diseases, underscoring the importance of considering both the quantity and quality of maternal contributions in early developmental processes and their long-term effects. Some maternal factors primarily function before MBT, but their phenotypic consequences only emerge post-MBT, while others persist beyond MBT, working alongside zygotic gene products for specific developmental processes [107,124,125].

Furthermore, when studying zygotic genes, maternal contributions can sometimes be misunderstood as a GCR or as mRNA degradation via NMD, if the potential maternal contribution is not taken into account. The variability in the amount of maternal products, including both positive (healthy mRNA) and negative (mutated mRNA) contributions, can cause a wide range of phenotypes among the offspring, as observed in maternal mutants with evident morphological defects after MBT in various organisms like flies, fish, and amphibians [106,107,108,109,110,126,127,128]. This phenotypic variability represents an opportunity to study human diseases and the effects of mutations at different developmental stages. The negative contribution of mutated maternal mRNA can affect maternal–zygotic genes involved in adult development, making them more prone to phenotypic changes. Intuitively, exclusively maternal genes lack genetic compensation due to the absence of early zygotic transcription, leaving the embryo dependent on maternal gene products. Conversely, exclusively zygotic genes cannot be compensated by maternal contributions, potentially leading to developmental issues. The theory of graded maternal contribution suggests that, while compensation between maternal and zygotic products can sometimes ensure the viability of homozygotes, they remain vulnerable to phenotypic alterations. This theory helps explain the phenotypic variability seen in mutant studies and highlights the importance of maternal genetic load in shaping the development and phenotype of progeny, with significant implications for studying genetic diseases and embryonic development [89,90,91].

### 4.2. Types of Maternal Contribution

Understanding maternal contributions and their effects is crucial for grasping how maternal genetic inputs shape embryonic development and influence compensatory mechanisms like the GCR. By categorizing maternal contributions into positive and negative types, and further dividing negative contributions into insufficiency or toxicity, we can better anticipate developmental outcomes and phenotypic variability, particularly regarding mutations that might either exacerbate or alleviate genetic disorders.

Positive Contribution: Refers to the maternal provision of WT mRNA and proteins that support normal development and ensure proper embryonic growth.

Negative Contribution: Involves the maternal provision of mutant mRNA, which can disrupt development. Negative contributions are further classified into:

Insufficiency: Occurs when levels of mutant mRNA or protein are too low to support normal development, leading to developmental defects.

Toxicity: Arises when mutant mRNA with PTCs escapes NMD and maternal clearance, leading to zygotic effects. Examples of maternal genes are not yet well-documented in the literature, but this remains a critical area for future research.

Examining how different mutations and maternal contributions affect embryonic development and GCR reveals key insights into phenotypic variability and disease outcomes. By distinguishing between positive and negative maternal influences, and understanding their impact on GCR, we can better grasp how mutations in maternal, zygotic, and maternal–zygotic genes shape developmental processes and phenotypic expression:

Maternal Gene: Nonsense mutations in maternal genes may struggle with early compensation due to lack of zygotic support, leading to severe defects. *Missense* mutations might cause milder effects if the altered protein retains some function.

Maternal–Zygotic Gene: Nonsense mutations may trigger genetic compensation after MZT, but early defects can arise if maternal contribution is insufficient. Missense mutations might be partially buffered by maternal products but could still cause issues during zygotic control.

Zygotic Gene: Nonsense mutations might activate genetic compensation, reducing defects, but failures in compensation can cause significant issues. *Missense* mutations may result in milder phenotypes if the protein retains partial function or compensation is effective.

Maternal–zygotic heterozygotes may exhibit severe phenotypes similar to those of homozygotes, but survival can be influenced by zygotic compensation through the GCR or by the mutation type. In cases like CHARGE syndrome, where GCR is not effectively activated, nonsense mutations can lead to severe outcomes, including potential lethality. However, this outcome is context-dependent and may vary based on the mutation type and the effectiveness of maternal and zygotic contributions. The understanding of these effects remains largely theoretical, particularly concerning the timing and mechanisms of transcriptional adaptation, and is especially relevant for genes that either escape or survive maternal clearance mechanisms.

### 4.3. Maternal Influence and Genetic Compensation in CHARGE Syndrome

CHARGE syndrome, driven by de novo mutations in the CHD7 gene, exemplifies the intricate interplay between PTCs, maternal influence, and genetic compensation mechanisms. Although *CHD7* is traditionally considered a zygotic gene, studies in zebrafish models have revealed a significant maternal effect on disease manifestation. This maternal contribution can significantly alter phenotypic expression, leading to notable variability among mutants with the same mutation, even in some cases where no observable phenotypes are present [115,116]. This duality raises important questions about the role of maternal genetic material in embryonic development and the phenotypic expression of *CHD7* mutations, necessitating the consideration of both zygotic and maternal effects in the study of CHARGE syndrome. 

About 78% of patients with *CHD7* mutations have nonsense and frameshift mutations that generate PTCs, while 11% have splice site mutations and 8% have missense mutations [13,129]. The disease in heterozygous individuals is typically attributed to haploinsufficiency of the gene, though this is questioned because these mutations are expected to trigger NMD. However, it is known that only a small fraction of mRNAs with PTCs actually undergo NMD in humans. When these PTC-bearing mRNAs escape degradation, they can accumulate and potentially trigger the disease. Notably, a case study involving CHARGE syndrome highlights this complexity, where a Japanese patient with a frameshift mutation in the *CHD7* gene developed PTCs. The authors suggested that even if the mutated transcripts escaped NMD, the truncated CHD7 protein would only contain the N-terminal chromodomains, being incapable of performing functions due to the loss of chromatin remodeling activity [20]. The persistence of such truncated proteins, which evade NMD, can result in the development of disease-associated phenotypes, highlighting the complex interplay between PTC positions, NMD activity, and genetic compensation.

The efficiency of NMD and its influence on phenotypic variability is evident in CHARGE syndrome. Mutations in the *CHD7* gene can lead to differences in DNA remodeling activity, which contribute to the phenotypic diversity observed among individuals [13,130]. The transmission of *CHD7* mutations from parents to offspring is rare, but when it does occur, parents often exhibit a mild or asymptomatic phenotype, while their children suffer from severe defects. This suggests that *CHD7* mutations primarily impact the maternal–zygotic stage, where maternal contributions may initially compensate for the mutation but are insufficient to prevent severe zygotic phenotypes [14,15,16,17,18,19]. Somatic and germline mosaicism further complicate this variability, as they can result in mutations being present in some cells but not others, which may explain why parents are often asymptomatic or have mild phenotypes, whereas their children, who inherit the mutation more uniformly, exhibit severe phenotypes [14,15].

The NMD pathway is crucial for eliminating mRNAs with PTCs to prevent the production of harmful truncated proteins. However, its efficiency varies significantly depending on the PTC’s location within the transcript. NMD is generally more effective when the PTC is before the last exon–exon junction but less effective in the first 200 nucleotides of the coding sequence or the last exon [118,119,120]. Not all PTCs are effectively targeted by NMD, particularly those in the last exon or near the end of the penultimate exon, which often escape degradation. This escape can lead to truncated proteins or trigger genetic compensation via homologous gene upregulation [71,72,118,120]. Such variability in NMD efficiency has significant implications for genetic diseases, as it contributes to the phenotypic diversity observed among individuals with the same mutation. PTCs from frameshift or nonsense mutations are particularly influential in this variability during embryonic development, especially when exacerbated by a graded maternal negative load. Notably, around 30% of human hereditary diseases are linked to PTCs or similar mutations introducing nonsense codons into mRNAs, with these mutations also frequently implicated in various cancers [130,131,132,133,134].

Moreover, the variability observed in zebrafish mutants, particularly those carrying PTCs in genes like *chd7*, reflects the delicate balance between mutation-induced toxicity and the organism’s compensatory mechanisms. The GCR to mutations varies based on factors such as developmental stage and the specific gene involved, influencing phenotypic outcomes. In zebrafish models, where genetic compensation plays a vital role in mitigating the effects of PTCs, the location of these codons within the gene significantly impacts the phenotype, mirroring the complexities observed in human genetic disorders [12,13,14,15,16,17,18,19,20,115,130,135,136]. This understanding underscores the importance of studying PTC-induced toxicity and genetic compensation in zebrafish as a model for developing therapeutic strategies for human diseases.

## 5. New Proposal for Genetic Classification

The proposal for a new genetic classification emerges from the need to integrate cellular mechanisms like the GCR and maternal contribution, both of which are crucial for phenotypic manifestation. Phenotypic recapitulation is vital for assessing the consistency between mutants and morphants, with significant overlap observed, despite some mutants only reproducing portions of the phenotypes in different tissues, suggesting potential spatial–temporal compensatory mechanisms [8,82,83,91,131]. The ability of mutant models to replicate morphant phenotypes emphasizes the significance of factors such as protein dysfunction, maternal contribution, and GCR activity in determining the final phenotype.

Based on described findings on phenotypic recapitulation already described in this article, we propose a new classification that categorizes genes into three types: susceptible genes, immune genes, and “Schrödinger” genes or conditional genes. This classification, rooted in the phenotypic recapitulation of morphants, emphasizes how well mutant models can reproduce morphant phenotypes and underscores the Schrödinger Paradox, illustrating how gene expression may lead to different phenotypes depending on specific experimental conditions. This new framework provides a fresh perspective for interpreting and designing genetic experiments in zebrafish by accounting for both genetic editing techniques and underlying biological mechanisms.

Susceptible Genes: These genes, whether maternal or zygotic, consistently present a phenotype because compensatory mechanisms do not effectively mitigate their mutations. Any alteration in their expression or protein structure directly impacts the phenotype, as these genes typically have low redundancy and minimal potential for rescue by maternal contribution or GCR. Mutations that significantly alter protein structure often result in clear, defective phenotypes. Mutations in genes like *gata2a*, *ccbe1*, and *flt4* often result in clear, defective phenotypes. *Gata2a* mutations cause severe circulation defects, *ccbe1* mutations disrupt lymphatic vessel development, and *flt4* mutations lead to the loss of key lymphatic structures, illustrating that neither genetic compensation nor maternal contributions can effectively compensate for these disruptions [8].

Immune Genes: These genes generally do not show a phenotype because compensatory mechanisms, such as GCR and maternal contributions, are highly effective in mitigating the effects of mutations. The concept of immune genes depends on the mutation being nonsense, producing PTCs that are typically detected by the NMD pathway, potentially triggering GCR. It is hypothesized that GCR could potentially occur in the germline, particularly in follicular cells. If this were the case, homozygotes for a maternal gene might activate compensatory genes, further diminishing phenotypic effects. However, this remains a theoretical possibility requiring further investigation. Immune genes would primarily be unaffected by missense mutations, as these mutations do not typically activate GCR or NMD. Examples of immune genes include *pdgfrb* and *nrp1a* [8]. For *pdgfrb*, phenotypes only arise with missense mutations, indicating strong genetic compensation for nonsense mutations. *nrp1a* shows incomplete phenotypes or less severe defects in mutants compared to morphants, suggesting effective compensation or off-target effects in morphants.

“Schrödinger” (Conditional) Genes: These genes, typically maternal–zygotic, are characterized by complex interactions between maternal and zygotic contributions. Schrödinger genes could be affected by both nonsense and missense mutations. However, maternal compensation might prevent the effects of missense mutations, and zygotic GCR could compensate for zygotic deficiencies caused by these mutations. The “Schrödinger Paradox” further illustrates that these genes may or may not exhibit a phenotype depending on the experimental conditions, emphasizing the unpredictable and context-dependent nature of gene expression outcomes. According to our hypothesis regarding immune genes and GCR in the germline, this compensation might also extend to the maternal contribution. Schrödinger genes exhibit ambiguous phenotypic effects due to the interplay of maternal buffering and zygotic GCR. Under certain conditions, these genes may not show a phenotype because compensatory responses effectively counteract the mutations. However, if these mechanisms are insufficient, a phenotype may emerge, making them appear conditionally phenotypic. This category reflects the complex balance required to maintain normal development, and while these genes could theoretically be considered immune genes if they manage to at least partially recapitulate the morphant phenotype depending on the extent of GCR and maternal contribution involved, it is appropriate to maintain a separate classification. Examples of Schrödinger genes might include *chd7* and *kpna7*. The *CHD7* gene, associated with CHARGE syndrome, can exhibit variable phenotypes depending on the balance between maternal contributions and genetic compensation mechanisms [115,116,135,136]. Similarly, *kpna7*, essential for nuclear import during early zebrafish development, could hypothetically exhibit a Schrödinger-like behavior. The presence of maternal transcripts might mask the effects of a *kpna7* mutation, leading to variable phenotypes depending on the extent of maternal contribution and genetic compensation [42].

However, while this model is valuable for understanding phenotypic discrepancies and gene categorization, it is crucial to recognize that poor experimental design—like inadequate MO controls or insufficient CRISPR validation—can lead to gene misclassification, underscoring the need for a rigorous methodology. The classification of genes into susceptible, immune, and Schrödinger categories arises from the concept of quantum appearances, which explain why not all genes can be universally classified as susceptible, immune, or Schrödinger. While it might seem that all genes are susceptible to phenotypic recapitulation, given their importance and observed outcomes across various mutagenesis techniques, this is not the case because phenotypic expression depends on factors such as the type of mutation, the nature of protein dysfunction, GCR activity, genetic redundancy, and maternal contributions [8,82,83,91,131]. 

Immune genes, like *pdgfrb*, generally do not exhibit a phenotype due to robust compensatory mechanisms; however, quantum appearances can occur when different mutations within the same gene lead to variable phenotypic outcomes, depending on whether the mutation disrupts these compensatory responses [8,137]. In contrast, Schrödinger genes, such as *chd7*, show phenotypic variability within the same mutation across different individuals, influenced by the complex interaction between maternal and zygotic gene products, which can either mask or reveal the phenotype depending on the genetic and environmental context. This distinction is particularly evident in Schrödinger genes, where genetically identical homozygous siblings, which supposedly receive the same maternal contribution, can display different phenotypes if the mother is heterozygous, due to the graded nature of maternal contributions. This phenotypic variability appears in both humans with CHARGE syndrome and *chd7* mutant zebrafish, among individuals with the same or different mutations [12,13,14,15,16,17,18,19,20,115,116,130,135,136]. Thus, precision in genetic research is essential to accurately interpret these phenomena, considering mutation type, developmental stage, gene interactions, and cellular environment, which all play a significant role in phenotypic expression and classification, ultimately guiding our understanding of gene function and disease mechanisms.

## 6. Conclusions

Our main conclusion, grounded in the theory of graded maternal contribution, posits that the uneven distribution of maternal products during gametogenesis introduces phenotypic variability that complicates the interpretation of results in knockout models. In the early stages of development, maternal contributions of RNA and proteins can mask the effects of mutations, leading to phenotypes that are less severe or different from those observed in morphants. This variability underscores the importance of considering graded maternal contribution as a key factor in phenotypic expression and in designing future studies aimed at better understanding gene function and compensation mechanisms in zebrafish development.

To enhance the accuracy of genetic research, it is imperative that the scientific community collaborates to improve the quality and accessibility of phenotypic and genetic databases. The establishment of collaborative and standardized platforms will simplify the interpretation of extensive datasets, enabling the quicker identification of genotype–phenotype relationships. We propose that the phenotypic differences observed between knockout and knockdown techniques in zebrafish are primarily influenced by graded maternal contribution rather than solely by GCR or NMD pathways.

Moreover, studying maternal genes by acquiring homozygous mutants or eliminating maternal RNA is essential for understanding these complex dynamics. Due to the intricate nature of genetic regulation, genes that involve both maternal and zygotic contributions will pose significant challenges for research. A deeper understanding of the relationship between the position of PTCs and the specific impact of GCR activation, alongside the maternal contribution, will be crucial. This knowledge will aid in developing more targeted and effective interventions, thereby improving the utility of zebrafish as a model in biomedical research and in the treatment of specific genetic diseases.

## Abbreviations

ENU	N-ethyl-N-nitrosourea
GCR	Genetic Compensation Response
MOs	Morpholino Oligos
MBT	Mid-Blastula Transition
MZT	Maternal–Zygotic Transition
NMD	Nonsense-Mediated Decay
PTC	Premature Termination Codon
TALEN	Transcription Activator-Like Effector Nucleases
WT	Wild Type
ZFN	Zinc Finger Nucleases
ZGA	Zygotic Genome Activation
